# Socioeconomic inequalities in uptake of HIV testing during antenatal care: evidence from Sub-Saharan Africa

**DOI:** 10.1186/s12939-023-02068-1

**Published:** 2024-01-08

**Authors:** Louis Kobina Dadzie, Aster Ferede Gebremedhin, Tarif Salihu, Bright Opoku Ahinkorah, Sanni Yaya

**Affiliations:** 1 Department of Population and Health, University of Cape Coast, Cape Coast, Ghana; 2 Cape Coast Teaching Hospital, Cape Coast, Ghana; 3 School of Public Health, Faculty of Health, University of Technology Sydney, Sydney, Australia; 4 School of International Development and Global Studies, University of Ottawa, Ottawa, K1N 6N5 Canada; 5 The George Institute for Global Health, Imperial College London, London, UK

**Keywords:** Socioeconomic inequalities, HIV testing, Antenatal care, Sub-Saharan Africa

## Abstract

**Background:**

Measuring socioeconomic inequalities in healthcare usage represents a critical step towards promoting health equity, in alignment with the principles of universal health coverage and the United Nations’ Sustainable Development Goals. In this study, we assessed the socioeconomic inequalities in HIV testing during antenatal care (ANC) in sub-Saharan Africa.

**Methods:**

Sub-Saharan Africa was the focus of this study. Benin, Burundi, Cameroon, Ethiopia, Gambia, Guinea, Liberia, Malawi, Mali, Mauritania, Mozambique, Rwanda, Sierra Leone, Uganda, Zambia, and Zimbabwe were the countries included in the study. This study used current Demographic and Health Surveys data spanning from 2015 to 2022. A total of 70,028 women who tested for HIV as part of antenatal contacts formed the sample for analysis. We utilized the standard concentration index and curve to understand the socioeconomic inequalities in HIV testing during antenatal care among women. Additionally, a decomposition analysis of the concentration index was ran to ascertain the contributions of each factor to the inequality.

**Results:**

Overall, 73.9% of women in sub-Saharan Africa tested for HIV during ANC. The countries with the highest proportions were Malawi, Rwanda, Zambia, and Zimbabwe. Mali Benin, Guinea, Mali, and Mauritania were the countries with the lowest proportions of HIV testing. Being among the richer [AOR 1.10, 95% CI: 1.02,1.18] and richest [AOR 1.41, 95% CI:1.30, 1.54] wealth quintiles increased the odds of HIV testing during ANC. The concentration value of 0.03 and the curve show that HIV testing is more concentrated among women in the highest wealth quintile. Hence, wealthy women are advantaged in terms of HIV testing. As the model’s residual value is negative (-0.057), the model overestimates the level of inequality in the outcome variable (HIV during ANC), which means that the model’s explanatory factors can account for higher concentration than is the case.

**Conclusion:**

We found that there is substantial wealth index-related inequalities in HIV testing, with women of the poorest wealth index disadvantaged in relation to the HIV testing. This emphasizes the necessity for sub-Saharan Africa public health programs to think about concentrating their limited resources on focused initiatives to grasp women from these socioeconomic circumstances. To increase women’s access to HIV testing, maternal and child health programs in sub-Saharan Africa should attempt to minimize female illiteracy and poverty. Consequently, health education may be required to provide women with comprehensive HIV knowledge and decrease the number of lost opportunities for women to get tested for HIV. Given the link between knowledge of HIV and HIV testing, it is important to focus on community education and sensitization about HIV and the need to know one’s status.

## Background

Despite the global acceleration of the AIDS response and recent medical advances, human immunodeficiency virus (HIV) remains a significant global health concern. According to the World Health Organization (WHO), HIV has caused the deaths of 40.1 million people to date and an estimated 38.4 million people were living with HIV at the end of 2021 [1]. Sub-Saharan Africa (SSA) is the epicenter of HIV-related morbidity and mortality. Approximately 25 million people in the region are living with HIV, accounting for two-thirds of the global total. Additionally, the region also accounts for over 70% of all AIDS-related deaths [1, 2].

Vertical transmission of HIV, which occurs from mother to child during pregnancy, childbirth, or breastfeeding, is a major contributor to the HIV epidemic in SSA, accounting for over 90% of new infections among children [3]. In the absence of any intervention, the transmission rate of HIV from an HIV-positive mother to her child can range from 15 to 45%. However, with effective intervention, the transmission rate can be lowered to below 5% [4]. The harmonization of essential approaches such as HIV education, availability of HIV testing services and support, early initiation and scale-up of antiretroviral therapy and Prevention of Mother-to-Child Transmission (PMTCT) programs can improve the course of pregnancy among HIV-positive mothers leading to safer mother-infant delivery and HIV-free infants [5]. PMTCT programs have been critical in reducing the incidence of new HIV infections in children in SSA. According to UNAIDS, the number of new HIV infections among children declined by 54% between 2010 and 2020 in the region. This was largely due to the expansion of PMTCT services and increased access to antiretroviral therapy for pregnant and breastfeeding women living with HIV [6].

Routine HIV testing during pregnancy enables early identification of HIV-positive pregnant women and the initiation of appropriate interventions to prevent mother-to-child transmission of HIV [7]. HIV testing services in SSA started with voluntary counselling and testing in standalone sites. With the expansion of antiretroviral therapy, provider-initiated testing and counselling emerged, and testing services were integrated into antenatal care, increasing coverage among pregnant and postpartum women [8, 9]. The joint United Nations Programme on HIV/AIDS has released a new set of goals to increase HIV testing and reduce new infections by 95% by 2030 [10]. However, HIV testing uptake among pregnant women remains suboptimal in sub-Saharan African countries [11, 12]. Studies have shown that access to HIV testing during antenatal care is often influenced by factors such as socioeconomic status, area of residence, and education level [12–18]. For example, it has been reported that the poorest and least educated population groups have lower rates of HIV testing. Socioeconomic factors are widely recognized to be driving forces behind the striking health inequalities in developing countries [19]. The level of socioeconomic inequalities in HIV testing during antenatal care in SSA has not been widely studied. Measuring socioeconomic inequalities in healthcare usage represents a critical step towards promoting health equity, in alignment with the principles of universal health coverage and the United Nations’ Sustainable Development Goals [20, 21]. In this study, we assessed the socioeconomic inequalities in HIV testing during antenatal care (ANC) in SSA.

## Methods

### Study area

SSA was the focus of this study. Specifically, 16 countries within the region (Benin, Burundi, Cameroon, Ethiopia, Gambia, Guinea, Liberia, Malawi, Mali, Mauritania, Mozambique, Rwanda, Sierra Leone, Uganda, Zambia, and Zimbabwe) were included in the study. These countries were chosen based on the availability of of variables considered in this study in their datasets as well as their survey years ranging from 2015 and beyond. 

### Data source and sampling

This study used the current Demographic and Health Surveys (DHS) of countries in SSA with data spanning from 2015 and beyond. The DHS provides comprehensive information on key issues including HIV and maternal and child health [22]. We used the women’s file for each country considered. 70,028 women with history of antenatal care visits formed the sample for analysis. The samples for the various countries have been presented in Table 1.

**Table 1:** Sample by countries

Country	Sample size	Percentage
Benin 2017-18	4,994	7.13
Burundi 2016-17	5,526	7.89
Cameroon 2018	4,682	6.69
Ethiopia 2016	4,864	6.95
Gambia 2019-20	3,377	4.82
Guinea 2018	3,373	4.82
Liberia 2019-20	2,266	3.23
Mali 2018	3,616	5.16
Mauritania 2019-21	4,780	6.83
Malawi 2015-16	7,586	10.83
Mozambique 2015	2,515	3.59
Rwanda 2019-20	4,665	6.66
Sierra Leone 2019	4,816	6.88
Uganda 2016	5,630	8.04
Zambia 2015	4,195	5.99
Zimbabwe 2015	3,143	4.49
All Countries	70,028	100

### Outcome variable

The outcome variable was HIV testing during ANC. To derive this variable, women aged 15 to 49 who had a live birth within the previous two years prior to the survey responded to the question on obtaining an HIV test result during ANC. This variable was coded as yes or no. DHS defined the prevalence of HIV testing during ANC as the proportion of women who had an HIV test during ANC and received the results along with a live birth two years before the survey.

### Explanatory variables

The key explanatory variable used for the decomposition was wealth index. In the DHS, the wealth index is constructed using household assets and ownership through principal component analysis as described in detail here and is comparable across all the survey years. The wealth index includes five quintiles (poorest, poorer, middle, richer, richest), where the first quintile stands for the less wealthy respondents. A number of other explanatory variables were considered for this study. Age was recoded as 15–19, 20–24, 25–29, 30–34, 35–39 and 40+. Level of education was also recoded as no education, primary, secondary or higher. Marital status was grouped into married and not married. Employment status was also categorized as working and not working. Assess to mass media was a composite variable created with the frequency of reading newspapers/magazines, frequency of listening to radio and frequency of watching television. The outcome was recoded as 0 if one had no access to at least of the above and 1 = if there was access. Comprehensive knowledge of HIV was a combination of a set of questions on the general knowledge of HIV transmission and misconception. Other variables in the study included the place of residence (rural or urban) [11].

### Analysis

Descriptive statistics assessed the data and provided frequencies and percentages of the variables considered. We used bivariate analysis to test the association between the explanatory variables and the outcome variable. Mixed effect logistic regression was applied to estimate the multivariable associations between the outcome and the explanatory variables. This allowed for the examination of both individual-level and group-level factors simultaneously and accounted for the clustering or nesting of observations within the higher-level units. We developed four models in total. The first model was a model with no explanatory variables included to estimate the intercept terms. The second model included only the individual-level factors which were age, level of education, marital status, occupation, media exposure and comprehensive knowledge of HIV. Next was the higher-level variables only. The higher-level factors considered were place of residence, wealth index and sub-region. Finally, all the variables were included for in the complete model. The estimation takes the basic form;

$$\begin{aligned}{\text{logit}}\,(\text{p}_{ij}) = \beta_{0} + \beta_{1}X_{1} + \beta_{2}X_{2} + ... + \beta_{\text{k}}X_{\text{k}} + \mu_{\text{j}} + \text{e}_{\text{ij}} \end{aligned}$$

Where:

Log(p_ij) is the log-odds of the outcome for the ith individual of the jth higher-level unit. β0 is the intercept, β1, β2, …, βk are the coefficients for the independent variables x1, x2, …, xk, u_j denotes the random effect for the jth higher-level unit and e_ij is the error term for the ith individual in the jth higher-level unit.

In our case, the formula for the final models can be written as;

### Model 0

$$\text{logit}\,({P_{ij}}) = \beta {0_j} + {e_{ij}}$$

### Model 1

$$\begin{aligned}\text{logit}\,(P_{ij}) = \beta 0_{j} + \beta 1* \text{age} + \beta 2* \text{education} + \beta 3* {\text{marital}_\text{status}}  \cr  + \beta 4* \text{occupation} + \beta 5* \text{media} + \beta 6* \text{knowledge}_{\text{on}_{HIV}} + \mu _{j} + e_{ij}\end{aligned}$$

### Model 2

$$\begin{aligned}{\text{logit}}\,({{\text{p}}_{\text{ij}}}) = \beta 0 + \beta 1*{\text{residence}} + \beta 2*{\text{wealth}}  \cr  + \beta 3*{\text{sub}}_{\text{region}} + \mu _{\text{j}} + {\text{e}}_{\text{ij}}\end{aligned}$$

### Model 3

$$\begin{aligned}\text{logit}\,({P_{ij}}) = \beta {0} + \beta {1}* {\text{age}} + \beta {2}* {\text{education}}  \cr  + \beta {3}* {\text{marital}_\text{status}} + \beta {4}* {\text{occupation}}  \cr  + \beta {5}* {\text{media}} + \beta 6* \text{knowledge}_{\text{on}_{HIV}}  \cr  + \beta {7}* {\text{residence}} + \beta {8}* {\text{wealth}}  \cr  + \beta {9}* {\text{sub}_\text{region}} + \mu _{j} + {e_{ij}}\end{aligned}$$

Where p is the probability of HIV testing during ANC, β0 is the intercept, and β1 to β9 are the coefficients for the independent variables age, level of education, marital status, occupation, media exposure, comprehensive knowledge of HIV, residence, wealth and sub-region respectively. All analyses were performed using STATA V.14 and catered for the compex survey design using weighting and the ‘svy’ command. With weight, first, individual country weighting was applied to the data before combining information from all countries. During this process, the standard weighting variable for the DHS (v005) was divided by 1,000,000, resulting in a new variable named “v005_pw.” Subsequently, the country-level weights were denormalized using the formula: v005_pwpool = v005_pw * (total population of women aged 15–49 at the time of the survey / number of women in the sample). Following the generation of country-level weights, data from the 16 countries were merged, and this dataset, along with the denormalized weights, was utilized for the final analysis.

### Concentration index

The concentration index is a widely used measure of socioeconomic inequality in health or healthcare utilization. It quantifies the degree of relative inequality across different population groups according to their socioeconomic status. In our study, we utilized the standard concentration index and curve to understand the socioeconomic inequalities in HIV testing during ANC among women belonging to different wealth index.

We followed the standard methodology widely employed in the literature to calculate the concentration index. We utilized a regression-based approach, specifically the concentration curve and associated concentration index. The corresponding concentration curve is a graphical representation of the cumulative distribution of the outcome variable (in our case, HIV testing during ANC) plotted against the corresponding cumulative share of the population ranked by wealth index. This allowed us to visualize the distribution of the HIV testing during ANC across different wealth index groups. The concentration index ranges from − 1 to + 1, with a value of zero indicating perfect equality, negative values indicating concentration among lower socioeconomic groups, and positive values indicating concentration among higher socioeconomic groups.

### Decomposition

Additionally, a decomposition analysis of the concentration index was run to ascertain the contributions of each factor to the inequality since the concentration index measures the overall inequalities in HIV testing during ANC among the wealth index of women. By decomposing the concentration index, we can identify and quantify the extent to which specific factors contribute to socioeconomic disparities in HIV testing. To perform the decomposition analysis of the concentration index, we used the standard decomposition of concentration indices. This technique helped to identify the relative contributions of different determinants or factors to the overall socioeconomic inequality captured by the concentration index.

## Results

**Fig. 1:**
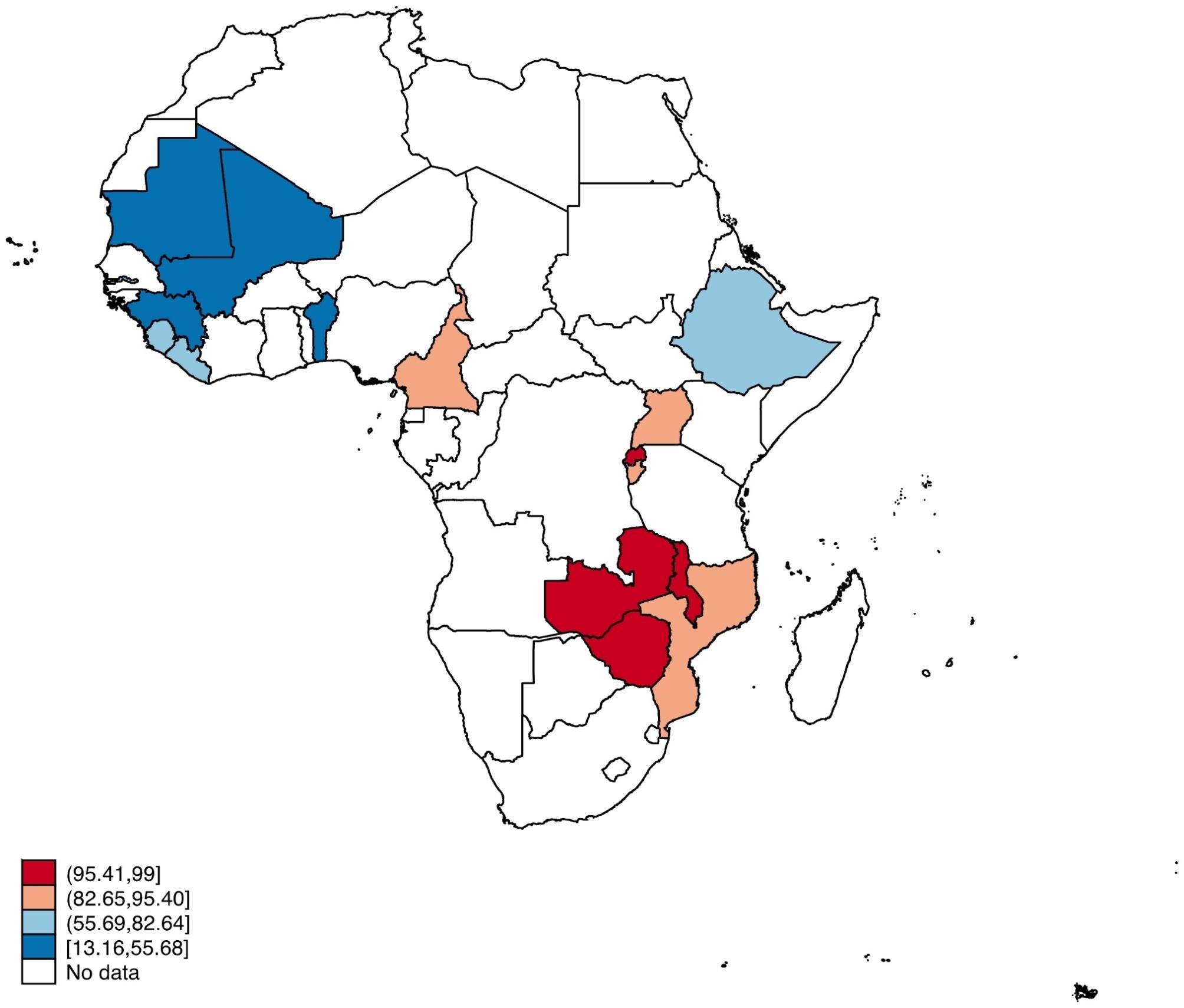
Spatial map showing the prevalence of HIV testing during antenatal visits

Figure 1 presents the prevalence of HIV testing during ANC for women across SSA. The countries with the highest proportions were Malawi, Rwanda, Zambia, and Zimbabwe. Mali Benin, Guinea, Mali, and Mauritania were the countries with the lowest proportions of HIV testing. Overall, 73.9% of women in SSA tested for HIV during ANC (Table 2).

It was observed that all the socio-demographic characteristics were significantly associated with HIV testing during antenatal visits. The highest proportion (26.2%) of women were aged 25–29 years and those aged 40 + were 6.7%. Approximately 39% of women had primary education and 69.2% lived in rural areas. More than a quarter (27.9%) of the women were not married. Women who were working represented 66.8%. More than half (56.1%) of the respondents had no comprehensive knowledge of HIV/AIDS. Women who had access to mass media were 63.3%. Respondents within the poorer category of the wealth index had the highest proportion (20.9%) and those in the richest had the lowest (18.2%) (see Table 2).

**Table 2:** Sociodemographic characteristics of women who tested for HIV as part of ANC

Variable	Freq (%)	Tested for HIV as part of ANC visit- Yes [95% CI]	P-value
**Tested for HIV as part of ANC visit**			
No	18,263(26.1)		
Yes	51,765(73.9)		
**Age**			< 0.001
15–19	6,440(9.2)	70.3 [68.5–72.0]	
20–24	17,233(24.6)	75.8 [74.6–77.0]	
25–29	18,313(26.2)	73.8 [72.5–75.1]	
30–34	13,832(19.8)	74.8 [73.5–76.0]	
35–39	9,494(13.6)	73.3 [71.9–74.7]	
40+	4,717(6.7)	71 [69.1–72.8]	
**Level of education**			< 0.001
No education	22,697(32.4)	56.1 [54.6–57.6]	
Primary	27,589(39.4)	80.6 [79.6–81.7]	
Secondary/Higher	19,743(28.2)	85 [84.0-85.9]	
**Place of residence**			0.038
Urban	21,546(30.8)	75.4 [73.8–77.0]	
Rural	48,482(69.2)	73.3 [72.0-74.4]	
**Marital status**			< 0.001
Not Married	19,536(27.9)	85.4 [84.5–86.3]	
Married	50,492(72.1)	69.5 [68.3–70.6]	
**Respondent’s occupation**			< 0.001
not working	23,246(33.2)	66.4 [64.8–68.0]	
Working	46,782(66.8)	77.6 [76.7–78.6]	
**Wealth index**			< 0.001
Poorest	14,653(20.9)	69.3 [67.5–71.0]	
Poorer	14,680(21)	70.6 [69.0-72.1]	
Middle	13,969(19.9)	71.8 [70.2–73.3]	
Richer	13,954(19.9)	76 [74.5–77.4]	
Richest	12,772(18.2)	83.1 [81.7–84.4]	
**Access to mass media**			< 0.001
No	25,732(36.7)	72.2 [70.8–73.5]	
Yes	44,296(63.3)	74.9 [73.9–76.0]	
**Comprehensive knowledge on HIV/AIDS**			< 0.001
No		64.6 [63.3–65.8]	
Yes		87 [86.2–87.7]	
**Sub-region**			< 0.001
Southern Africa	17,439(24.9)	95.3 [94.6–95.8]	
Western Africa	22,440(32)	55.3 [53.7–56.8]	
Eastern Africa	15,160(21.6)	84.4 [82.3–86.3]	
Central Africa	10,209(14.6)	91.3 [90.1–92.4]	
North Africa	4,780(6.8)	13.2 [11.2–15.3]	

From Table 3, the odds of HIV testing during ANC increased with age. For instance, women who were 40 + years were more likely [AOR 2.00, 95% CI: 1.73, 2.31] to test for HIV during ANC compared to women aged 15–19 years. Married women had significantly lower odds of HIV testing during ANC than those who were not married [AOR 0.60, 95% CI: 0.55, 0.66]. Women were more likely to test for HIV during ANC if they were working [AOR 1.47, 95% CI: 1.36, 1.59] and had comprehensive knowledge of HIV/AIDS [AOR 1.95, 95% CI: 1.81,2.10] compared to women who did not work and those who did not have comprehensive knowledge of HIV/AIDS, respectively. Level of education increased the odds of HIV testing during ANC, with women with secondary/higher education more likely to test for HIV during ANC compared to those with no formal education [AOR 2.71, 95% CI: 2.47, 2.98]. Women who lived in rural areas were less likely to test for HIV during ANC relative to their counterparts in the urban areas [AOR 0.64, 95% CI: 0.56, 0.74]. Being among the richer [AOR 1.19, 95% CI: 1.05,1.34] and richest [AOR 1.47, 95% CI: 1.26, 1.72] wealth quintiles increased the odds of HIV testing during ANC. HIV testing during ANC among women who had access to mass media was significantly lower [AOR 1.18, 95% CI: 1.09, 1.28] than those who had no access to mass media.

**Table 3:** Mixed effects logistic regression of factors associated with HIV testing during ANC

Variable	Model 0	Model 1	Model 2	Model 3
**Age**				
15–19		Ref		Ref
20–24		1.34*** [1.23,1.47]		1.35*** [1.22,1.50]
25–29		1.41*** [1.29,1.55]		1.56*** [1.40,1.74]
30–34		1.66*** [1.49,1.84]		1.81*** [1.61,2.04]
35–39		1.66*** [1.48,1.85]		1.86*** [1.64,2.11]
40+		1.72*** [1.52,1.94]		2.00*** [1.73,2.31]
**Level of education**				
No education		Ref		Ref
Primary		3.05*** [2.84,3.28]		1.79*** [1.65,1.94]
Secondary/Higher		4.19*** [3.83,4.58]		2.71*** [2.47,2.98]
**Marital status**				
Not Married		Ref		Ref
Married		0.47*** [0.43,0.50]		0.60*** [0.55,0.66]
**Respondent’s occupation**				
Not working		Ref		Ref
Working		1.76*** [1.65,1.88]		1.47*** [1.36,1.59]
**Access to mass media**				
No		Ref		Ref
Yes		0.86*** [0.80,0.92]		1.18*** [1.09,1.28]
**Comprehensive knowledge on HIV/AIDS**				
No		Ref		Ref
Yes		2.92*** [2.73,3.12]		1.95*** [1.81,2.10]
**Place of residence**				
Urban			Ref	Ref
Rural			0.55*** [0.48,0.63]	0.64*** [0.56,0.74]
**Wealth index**				
Poorest			Ref	Ref
Poorer			1.15** [1.05,1.27]	1.07 [0.98,1.18]
Middle			1.32*** [1.19,1.46]	1.15** [1.04,1.28]
Richer			1.49*** [1.32,1.68]	1.19** [1.05,1.34]
Richest			2.24*** [1.93,2.61]	1.47*** [1.26,1.72]
**Sub-region**				
Southern Africa			Ref	Ref
Western Africa			0.04*** [0.03,0.05]	0.06*** [0.05,0.07]
Eastern Africa			0.28*** [0.23,0.33]	0.25*** [0.21,0.30]
Central Africa			0.49*** [0.40,0.60]	0.45*** [0.37,0.55]
North Africa			0.03*** [0.01,0.04]	0.02*** [0.01,0.03]
N	70,028	70,028	70,028	70,028
AIC	246448.67	213502.26	180448.59	170473.86
BIC	246466.98	213621.3	180549.31	170675.31
log likelihood	-123222.34	-106738.13	-90213.295	-85214.93

Exponentiated coefficients; 95% confidence intervals in brackets; Ref = Reference

* p < 0.05, ** p < 0.01, *** p < 0.001

**Fig. 2:**
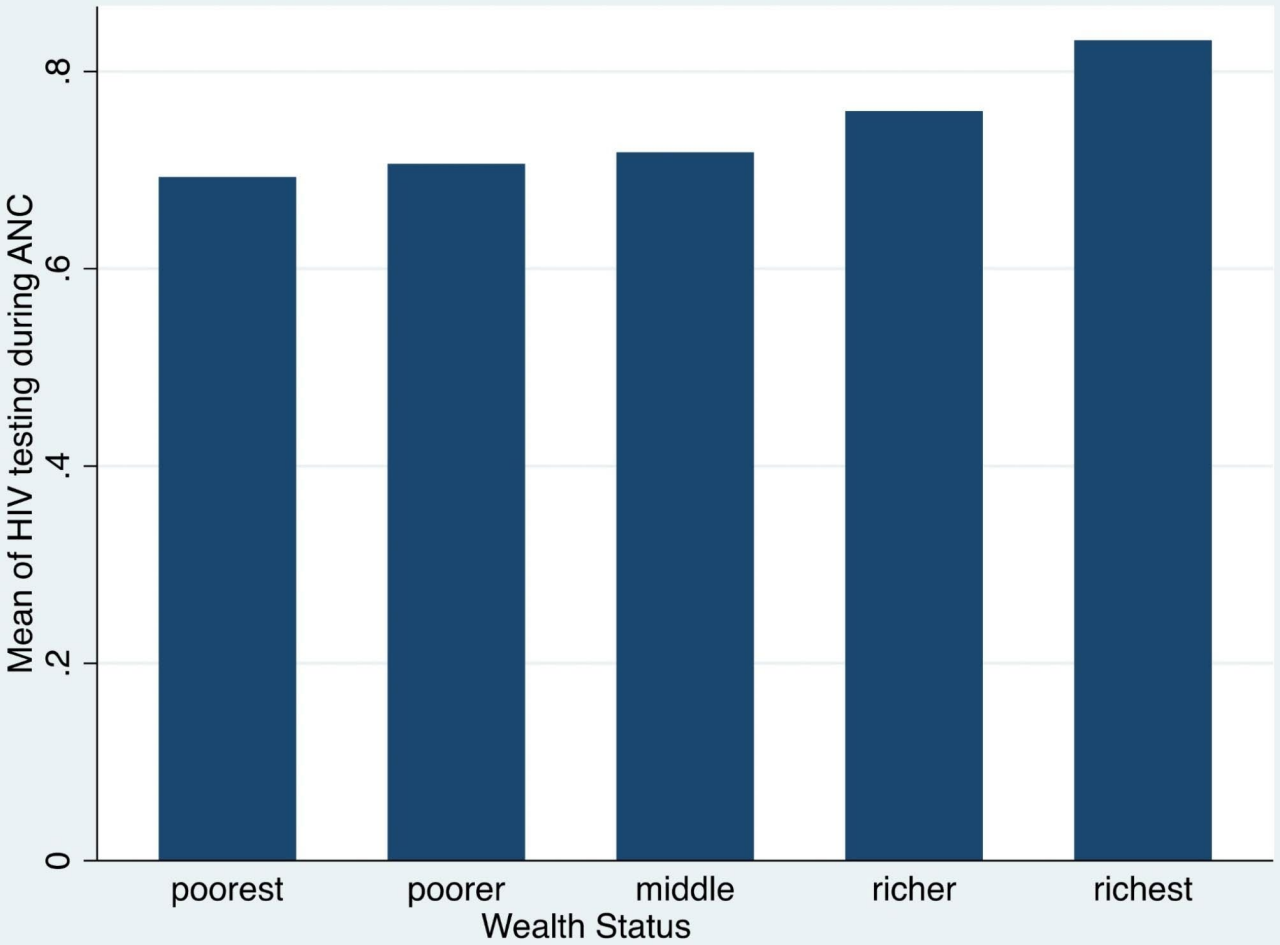
Mean of testing during ANC across wealth status

Figure 2 indicates the distribution of HIV testing during ANC across wealth index. The results indicate that the mean distribution of HIV testing during ANC increases with wealth index, with the highest mean among women of the richest wealth index and the lowest mean among those of the poorest wealth index.

**Fig. 3:**
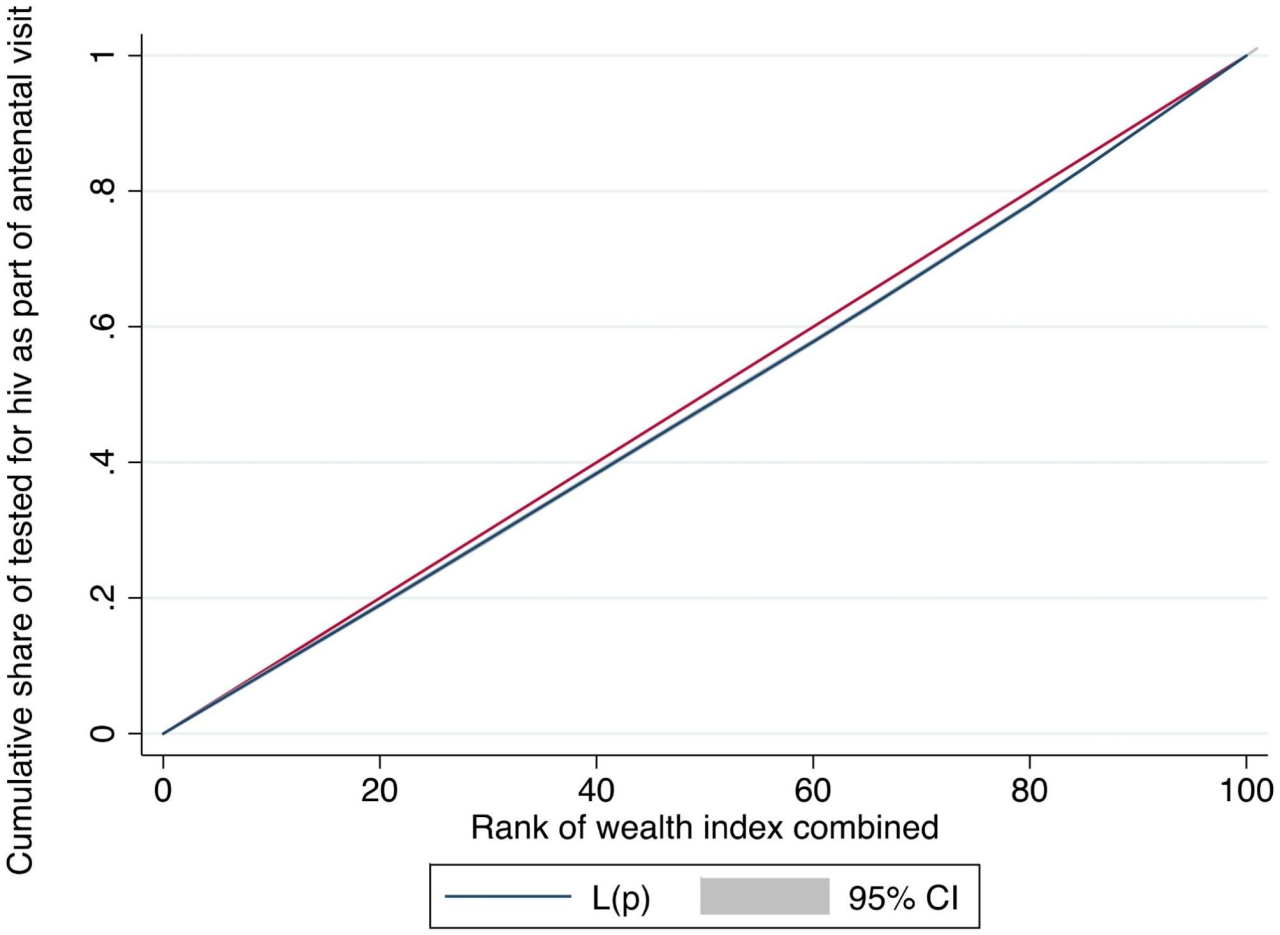
Concentration Curve of HIV testing during ANC by wealth status

Figure 3 indicates that HIV testing during ANC was concentrated in an affluent class of women as realized from the curve which lies below the equality line. The concentration value of 0.03 and the curve show that the HIV testing is more concentrated among women in the highest wealth quintile. Hence, wealthy women are advantaged in terms of HIV testing. The relative weights of each component in explaining the observed inequality are shown in Table 4. The percentage contribution denotes the proportion of overall inequality that each factor is responsible for. If a factor has a positive percentage contribution, it is increasing the observed inequality; if a factor has a negative percentage contribution, it is decreasing the observed inequality. 46.3% of the inequalities seen for HIV testing during ANC are caused by secondary and higher-level education. Contrarily, variables like marital status and media access lessen inequality by 32% and 10%, respectively. As the model’s residual value is negative (-0.057), it is clear that the model overestimates the level of inequality in the outcome variable (HIV during ANC), which means that the model’s explanatory factors can account for higher concentration than is the case (see Table 5).

**Table 4:** Concentration index by sub-regions

Sub-regions	No. of obs.	Index value	Std. error	p-value	
All countries	70,028	0.03465532	0.00293537	< 0.001	
Southern Africa	18,812	0.00993615	0.0013398	< 0.001	
Western Africa	20,747	0.0858076	0.00637832	< 0.001	
Eastern Africa	15,054	0.03293851	0.00445816	< 0.001	
Central Africa	10,765	0.02296114	0.0028688	< 0.001	
North Africa	4,650	0.33261418	0.03790364	< 0.001	

Test for stat. significant differences with Ho: diff = 0 (assuming equal variances)

F-stat = 281.77978, p < 0.000

**Table 5:** Decomposition analysis of the contribution of factors associated with the inequalities of HIV testing during ANC

Variable	Elasticity	Concentration index	Absolute contribution	% contribution	
**Age**					
15–19	Ref				1.449
20–24	0.033	-0.015	-0.001	-0.557	
25–29	0.052	0.040	0.002	2.331	
30–34	0.051	0.027	0.001	1.500	
35–39	0.038	0.000	0.000	-0.005	
40+	0.020	-0.083	-0.002	-1.820	
**Level of education**					
No education	Ref				35.413
Primary	0.105	-0.093	-0.010	-10.892	
Secondary/Higher	0.120	0.347	0.042	46.305	
**Place of residence**					
Urban	Ref			.	28.903
Rural	-0.124	-0.209	0.026	28.903	
**Marital status**					
Not Married	Ref			.	-0.319
Married	-0.151	0.002	0.000	-0.319	
**Respondent’s occupation**					
Not working				.	-4.237
Working	0.121	-0.032	-0.004	-4.237	
**Wealth index**				.	
Poorest	Ref			.	33.317
Poorer	0.006	-0.372	-0.002	-2.632	
Middle	0.009	0.037	0.000	0.375	
Richer	0.013	0.436	0.006	6.472	
Richest	0.032	0.818	0.026	29.102	
**Access to mass media**					
No	Ref				6.356
Yes	0.038	0.152	0.006	6.356	
**Comprehensive knowledge on HIV/AIDS**					
No	Ref				12.336
Yes	0.127	0.087	0.011	12.336	
**Sub-region**					
Southern Africa	Ref				-12.724
Western Africa	-0.369	0.019	-0.007	-7.818	
Eastern Africa	-0.118	0.017	-0.002	-2.224	
Central Africa	-0.054	-0.004	0.000	0.225	
North Africa	-0.144	0.018	-0.003	-2.908	
Calculated CII			0.09		
Actual CII			0.034		
Residual			-0.056		

Ref = reference

## Discussion

The socioeconomic inequalities in HIV testing during ANC in SSA were assessed in this study. This study demonstrated that there is greater socioeconomic inequality in HIV testing during ANC contacts in SSA among the wealthy than the poor. According to this study’s decomposition analysis, socioeconomic characteristics such as age, education level, marital status, occupation, place of residence, wealth index, access to the media, comprehensive knowledge on HIV/AIDS, and sub-region were found to be considerably connected with inequalities in HIV testing during ANC contacts in SSA. The most significant determinants of overall inequality in HIV testing thru ANC contacts among women in SSA were education level, place of residence, wealth index, access to mass media, and comprehensive knowledge on HIV/AIDS.

According to analyses done per country, Rwanda had the highest prevalence of HIV testing (99%), followed by Zimbabwe (98.22%) and Zambia (97.63%), respectively. Nonetheless, Mauritania (13.16%) had the lowest incidence. The highest incidence witnessed in Rwanda could be attributed to the deployment of countrywide campaigns to promote HIV testing and counselling (HTC), as well as the operative and competent implementation of PMTCT to certify that the majority of women are tested for HIV and those detected are put on medication (Gazimbi et al., 2019; Awopegba et al., 2020). It might also be a result of the Rwanda Ministry of Health’s endorsement of provider initiated testing and counselling (PITC) as a strategy to expand the availability of HIV testing and warrant prompt HIV diagnosis among women (Awopegba et al., 2020; Kayigamba et al., 2015). The lowest HIV testing rate found in Mauritania is, however, far lower than the rates reported in earlier research, which included 35.1% in Ethiopia [25], 35.1% in Mozambique [26], 81.5% in Uganda [11], and 30% in India [27]. The low prevalence could be linked to the low uptake of ANC services in Mauritania, as women in West Africa were generally less probable to obtain ANC [12]. The discrepancies in rates could be the result of various variances in sample sizes, study periods, and research techniques. It could also be because these studies use different definitions of HIV testing during prenatal care [28]).

This study found inequalities in the HIV testing of women in SSA during ANC based on socioeconomic characteristics. It is commonly known that the SDGs support reducing inequality and ensuring everyone has access to health care. Given the aforementioned, it is vital to investigate how to reach the most disadvantaged group of women through the use of maternal health services, including HIV testing thru ANC contacts for mothers, in order to accomplish the objectives [29]. Earlier studies suggested that people’s socioeconomic situations and the characteristics of the users were obstacles to HIV testing during ANC [13, 25, 28]. Also, studies from low-and-middle-income countries showed that the cost of testing for HIV among women who are of reproductive age was a major impediment [25, 28]. As it was expected, we observed significant pro-rich inequalities in the HIV testing at ANC for SSA women. This translates to an upsurge in HIV testing as the wealth index grew from the poorest to the affluent categories. Specifically, socioeconomic level significantly influenced the number of women who had HIV testing during ANC contacts. This finding demonstrated how poverty greatly affects HIV testing thru ANC. Our findings, which indicate that there is discrimination against poorer populations when it comes to HIV testing during ANC are consistent with those of research conducted in the SSA [24], and India [27].

The increasing wealth-related inequality in HIV testing may be related to the widening affluence gaps across several SSA countries [38]), as well as the slow rate of poverty reduction in these countries [23]. The growing income inequality may put the affluent in a better position to obtain critical information via television, radio, and schools. Wealth-related inequalities in HIV testing at ANC have the effect of making the wealthy more knowledgeable than the poor [23]. As a result, the poor may find it more difficult to attend ANC, which could ultimately result in low uptake of HIV testing. Pro-rich economic inequality with regard to HIV testing continues across genders and regions [23]. The pro-rich inequality in HIV testing among women thru ANC contacts is a major concern and has policy consequences considering that free maternal health services, including PMTCT services, have been adopted in the majority of SSA countries (Astawesegn et al., 2022). This demonstrates that lowering financial barriers to increase access among various socioeconomic groups does not always translate into equitable service use. Even if the services do not require payment from patients, the region’s poor women may still have trouble accessing them due to transportation costs, a lack of services in some areas, lower levels of education, and inadequate comprehensive knowledge of HIV/AIDS (Astawesegn et al., 2022). Hence, one key proposal is to establish systems to assist the financially vulnerable in paying for all out-of-pocket expenses associated with receiving an HIV test (Khin et al., 2023).

This study established that women’s level of education significantly contributed to the HIV testing inequalities observed during ANC visits. It is worth noting that the results of our decomposition analysis showed that the education level of women alone was responsible for nearly (46.3%) of the inequalities observed for HIV testing during ANC. As education levels rise, so does the frequency of HIV testing thru ANC. As a result, women who are educated are more probable to test for HIV during ANC. Our findings support earlier research showing socioeconomic differences in HIV testing thru ANC in numerous African countries, including Ethiopia [25, 30], and Mozambique [26]. This result raises the possibility that mothers who are educated could be better aware of MTCT for HIV and comprehend the advantages of HIV testing at ANC. Moreover, educated mothers might be greatly exposed to information about HIV, comprehend the advantages of HIV therapy, and be better equipped to decide whether or not to get tested [27]. This study supports previous findings from other developing countries that women who have completed secondary school or higher are probable to know highly about MTCT [31]. In order to reduce inequality and boost SSA women’s use of HIV testing and PMTCT, it is crucial that female children receive better education. Governments and other stakeholders in the SSA countries ought to implement policies to increase the percentage of females who stay in school. One such option is to permit females who leave school early due to pregnancy to return and finish their studies after giving birth [31].

The findings of this study showed rural-urban inequality in HIV testing during ANC contacts among women in SSA. Comparing rural and urban women in SSA, it was found that the former had lower probability of testing for HIV thru ANC contacts. This illustrates a pro-urban inequality in the HIV testing thru ANC contacts. That is, there is a substantial disparity in HIV testing in favour of urban women and relatively less HIV testing among rural women. The rurality hypothesis may be influenced by a number of factors, including the fact that poor rural women frequently travel long distances to access medical facilities and may not be able to afford the transportation costs, as well as the possibility that harvesting activities may restrict their movement at certain times of the year [32]. These findings are in line with a large body of research from the SSA, which shows that the rural poor population has unequal access to HIV testing through ANC contacts [32, 33] and is more at risk of not using maternal health services, including HIV testing during ANC contacts in the SSA [34]. Women who lived in rural areas were undeservedly impoverished and accounted for roughly 29% of the overall inequality observed in this study. Despite the fact that services are provided for free, rural women still experience poor quality and weak integration of PMTCT services with maternal and child health services, poor roads, and greater difficulty accessing health services [35]. Higher rates of unemployment, lower levels of education, and greater poverty are characteristics of rural communities. In contrast, urban environments may offer greater employment opportunities, easier access to higher education, and successful PMTCT programme implementation in terms of service quality, availability, and integration [36]. Hence, outspreading the home or community-based HIV testing and counselling programme, offering mobile testing, and allowing for self-testing for HIV would increase the accessibility of the services to rural residents in rural regions, which could help to advance access and subsequently reduce inequality in HIV testing [37, 38].

### Strengths and limitations

The use of extensive, nationally representative surveys in this study is its key strength since it can help programmers and policymakers create operative national and regional intervention programs by giving them valuable information. Also, the data was carefully scrutinized to produce the study’s findings, and we used higher-order statistical approaches to do so.

Our study had very few restrictions. First, the study’s conclusions relied on self-reported results. The information provided by self-reports may be impacted by recall and reporting bias. Since that precision differs subject to HIV status, it might be challenging to evaluate the legitimacy of self-reported HIV tests. Variations in self-reporting precision between socioeconomic categories may have skewered our results due to the fact that inequality evaluations depend on quantifying a connection [13]. Second, it was impossible to tell from the DHS data if women had accepted or rejected an HIV test when it had been provided to her during ANC, which might have been related to socioeconomic level.

## Conclusion

This study assessed the socioeconomic inequalities in HIV testing during ANC for women in SSA. Our findings indicated that there is greater socioeconomic inequality in HIV testing during ANC contacts in SSA among the wealthy than the poor. Socioeconomic factors such as age, education level, marital status, occupation, wealth index, access to mass media, comprehensive knowledge of HIV, and sub-region substantially influenced differences in HIV testing thru ANC in SSA. The biggest contributors to the overall inequality in HIV testing observed in this study were level of education, place of residence, wealth index, access to mass media, and comprehensive knowledge on HIV/AIDS. This emphasizes the necessity for SSA public health programs to think about concentrating their limited resources on focused initiatives to grasp women from these socioeconomic circumstances. In order to increase women’s access to HIV testing and consequently eliminate inequality, MCH programs in SSA should try to minimize female illiteracy and poverty. Consequently, health education may be required to provide women with comprehensive HIV knowledge and decrease the number of lost opportunities for women to get tested for HIV. Given the link between knowledge of HIV and HIV testing, it is important to focus on community education and sensitization about HIV and the need of knowing one’s status.To address socioeconomic inequalities in HIV testing at ANC, governments in SSA should develop and support initiatives to keep females in school and fortify educational edifices to enhance admission to general secondary and tertiary education among women without education. This is due to the possibility that women’s educational status may be linked to increasing HIV testing during ANC. A particular financial support program for low-income and underprivileged women should be implemented by various governments throughout SSA. Such a plan might be designed to benefit economically vulnerable women by lowering their ANC and transportation costs. Also, to increase HIV testing and decrease inequities in ANC of HIV tests, it would also be beneficial to send targeted messaging to raise HIV awareness and knowledge among older women, low-income women, and the uneducated. These can help increase ANC coverage of HIV tests and decrease socioeconomic inequalities in HIV testing among women in SSA.

## Data Availability

The datasets generated and/or analyzed during the current study are available in http://dhsprogram.com/data/available-datasets.cfm.
